# Meconium Exposure to Phthalates, Sex and Thyroid Hormones, Birth Size and Pregnancy Outcomes in 251 Mother–Infant Pairs from Shanghai

**DOI:** 10.3390/ijerph17217711

**Published:** 2020-10-22

**Authors:** JiaLin Guo, Min Wu, Xi Gao, JingSi Chen, ShuGuang Li, Bo Chen, RuiHua Dong

**Affiliations:** 1 Shanghai First Maternity and Infant Hospital, Tongji University School of Medicine, Shanghai 200032, China; guojialin78@hotmail.com; 2 Key Laboratory of Public Health Safety of Ministry of Education, Collaborative Innovation Center of Social Risks Governance in Health, School of Public Health, Fudan University, Shanghai 200032, China; wumin@shmu.edu.cn (M.W.); 12211020018@fudan.edu.cn (X.G.); 15211020022@fudan.edu.cn (J.C.); leeshuguang@fudan.edu.cn (S.L.); chenb@fudan.edu.cn (B.C.)

**Keywords:** phthalates, birth size parameters, pregnancy outcomes, sex hormone, thyroid hormone

## Abstract

Phthalates are hormonally active pollutants. In-utero exposure to phthalates has been reported to be associated with birth size parameters and pregnancy outcomes. However, previous reports were inconsistent. We examined the associations between meconium exposure to phthalates and the effects on birth size parameters, pregnancy outcomes and sex and thyroid hormones in 251 mother–infant pairs from a Shanghai hospital. We measured 10 metabolites of phthalates in meconium samples collected during the first 24h after delivery. Information on seven birth size parameters (birth weight, birth length, abdominal circumference, head circumference, femur length, biparietal diameter and anogenital distance) and three pregnancy outcomes (gestational diabetes, premature rupture of membrane, and premature birth) was available from the birth record. Concentrations of free testosterone, estradiol (E2), thyroid stimulating hormone, concentrations of total and free thyroxine and triiodothyronine were measured from cord blood. Multivariate linear regression and logistic regression were used to estimate associations between phthalate exposure and health outcomes. mono-iso-butylphthalate (MiBP), mono-n-butylphthalate (MnBP) and mono-2-ethyl-5-oxohexyl phthalate (MEOHP) were positively associated with birth length and femur length which seemed more obvious in female newborn; MiBP, MnBP and mono-2-ethylhexylphthalate (MEHP) were positively associated with gestational diabetes mellitus (GDM) only in mothers with male newborns; monomethyl phthalate (MMP), MiBP and MEOHP were positively associated with E2 in male newborns. This study indicates that meconium exposure to phthalates may adversely affect some fetal growth parameters and GDM with a potential gender effect.

## 1. Introduction

Phthalates are environmental endocrine disruptors (EEDs) that are widely used in daily consumer products, resulting in ubiquitous human exposure [1,2]. While adult exposure to these chemicals is of importance, the exposure of fetuses and/or infants is of primary concern since those groups are extremely sensitive to the effects caused by phthalates with endocrine disruption properties [3,4]. Growing evidence suggests that in-utero exposure to phthalates may result in adverse birth outcomes, including the reduction in anogenital distance (AGD), weight and length at birth, and gestational age, as well as the increased risk of preterm delivery [5,6,7,8]. However, previous findings on these correlations are still controversial. Such controversy may result from different levels of exposure, limit sample size and unresolved confounders. Moreover, such association may be gender distributed as animal studies have presented a gender-specific effect of phthalates on fetal development [9,10], while this phenomenon has not been reported in human data.

Hormonal production and regulation are critical for pregnancy maintenance and fetal growth. Hormonal disruption has been considered as a potential mechanism through which exposure to phthalates adversely affects birth or pregnancy outcomes. Phthalates can interfere with the concentrations, signaling, and/or functions of sex hormones in rodent models [11], which may profoundly affect the implantation, development and parturition of fetus [12,13]. Human studies suggested that exposure to phthalates altered reproductive hormone levels in adults [14,15,16], while exposure during in utero development has been associated with changes in hormone levels in newborns [17]. For example, prenatal exposure to dis (2-ethylhexyl) phthalate (DEHP) was found to be negatively associated with free testosterone (FT) and total testosterone (TT) concentrations in pregnancy and in umbilical cord blood [18]. Additionally, experimental studies suggested that phthalate exposure may adversely influence thyroid homeostasis [14]. Epidemiologic studies also suggested that phthalates may adversely affect thyroid hormones among adolescents and adults, pregnant women, and newborns [19,20,21,22]. However, the direction and the implicated phthalates were inconsistent across studies.

It should be noted that maternal–fetal–infant transfer of phthalates occurs during gestation [23]. Most of the previous studies examined the associations between pregnancy exposure and sex or thyroid hormone levels during gestation in maternal matrices [24,25]. Measuring phthalate metabolites in newborn matrices rather than maternal matrices will assist with estimating the actual exposure for the fetus. However, few studies have examined the exposures of newborns and the effects of phthalates on hormone levels of newborns in their matrices directly. While several biomonitoring studies of phthalates in newborns that measured exposure by analysis of blood, urine and breast milk have been published, there is a paucity of data that have examined the newborns exposures in meconium [26]. Meconium has shown some promise as a matrix for evaluating accumulated exposure to phthalates in fetuses [27]. Herein, we chose the meconium level as representative of in-utero exposure to phthalates. Furthermore, none of the previous studies examine the exposure to phthalates, sex and thyroid hormone levels, and birth outcomes simultaneously. It remains unclear whether the associations between in-utero exposure to phthalates and birth and pregnancy outcomes are related to the disruption of sex or thyroid hormone levels. 

The aim of this study was to conduct a comprehensive study to examine the associations between meconium exposure to phthalates, sex and thyroid hormones, and birth size parameters and pregnancy outcomes in 251 mother–infant pairs in Shanghai, China. Furthermore, we explored whether or not these associations might be modified by a newborn’s gender.

## 2. Material and Methods

### 2.1. Study Population

We conducted a cross-sectional study in an obstetrical and gynecological hospital from Shanghai from July 2013 to July 2014. We recruited 258 women at the third trimester of pregnancy to participate in our study. Information on delivery and pregnancy characteristics including mother’s age, gestational age, gender of newborn and pregnancy outcomes were available from the birth record. For each newborn, meconium was collected directly from every diaper during the first 24 hours after delivery [26,28]. Excluding 7 cases that did not provide the meconium samples, 251 subjects with meconium and cord blood specimens were included in the present study. The study was undertaken with the permission of the local authority and the Ethics Committees of School of Public Health, Fudan University, PR China. Informed consent was obtained from each participant.

### 2.2. Measurement of Pregnancy Outcomes and Birth Size Parameters

Pregnancy outcomes included premature rupture of membrane (PROM), gestational diabetes mellitus (GDM) and preterm delivery. GDM was diagnosed using a one-step approach with a 75g 2h-OGTT: at fasting ≥ 90 mg/dL, at 1 h ≥ 180 mg/dL, and at 2 h ≥ 153 mg/dL, according to the guidelines of the International Association of Diabetes and Pregnancy Study Groups [29]. PROM was defined as the rupture of the amniotic sac before the onset of labor. Preterm birth was defined as the birth of a baby at fewer than 37 weeks’ gestation. We excluded the iatrogenic preterm birth caused by other factors, including placenta previa, fetal growth restriction and preeclampsia.

Birth size parameters included birth weight (BW), birth length (BL), head circumference (HC), abdominal circumference (AC), biparietal diameter (BPD), femur length (FL) and anogenital distance (AGD). Measurements of BW and BL were measured by clinical nurses, HC, AC, BPD, FL and AGD were based on standardized clinical techniques. 

### 2.3. Determination of Phthalate Metabolites in the Meconium Samples

Meconium from each infant was collected in glass tubes capped with polypropylene lids. Both tubes and lids had been previously washed to remove the background phthalates. Ten major monoester metabolites, namely monomethyl phthalate (MMP), mono ethyl phthalate (MEP), mono-n-butylphthalate (MnBP), mono-iso-butylphthalate (MiBP), mono benzyl phthalate (MBzP), mono-2-ethylhexylphthalate (MEHP), mono-2-ethyl-5-hydroxyhexyl phthalate (MEHHP), mono-2-ethyl-5-oxohexyl phthalate (MEOHP), mono (2-ethyl-5-carboxypentyl) phthalate (MECPP) and mono (2-carboxymethylhexyl) phthalate (MCMHP) were analyzed using the method modified by Kato et al. (2006) [30]. These metabolites were monoesters of 5 phthalate diesters, that is MEP from diethyl phthalate (DEP), MMP from dimethyl phthalate (DMP), MnBP and MiBP from dibutyl phthalate (DBP), MBzP from benzyl butyl phthalate (BBzP), and MEHP, MEHHP, MEOHP, MECPP and MCMHP from DEHP. Of the selected 5 phthalate diesters, DBP, DEP and DMP are three major low molecular weight (MW) phthalates being frequently used as solvents in personal care products, insecticides, lacquers and products, and DEHP and BBzP are two major high MW phthalates being frequently used in construction materials and numerous polyvinyl chloride (PVC) products [31]. The production and importation of phthalates in China has been reported to be dominated with DEHP and DBP [32]. All the selected 10 metabolites have been frequently detected in maternal urines in previous studies [26,32].

The analytical reference standards and internal standards were purchased from Cambridge Isotope Laboratories (CIL, USA). Briefly, 0.5g of meconium sample was incubated with β-glucuronidase at 37 °C for 90 min. The sample was subsequently acidified with 2 mL of methanoic acid (0.1mol/L), mixed with 50 μL of internal standard (100 μg/L), and loaded into a PLS column previously activated with 2 mL methanol and 2 mL of aqueous 0.5% (v/v) acetic acid. After sample loading, the column was washed and eluted with 0.5 mL of methanol and 2 mL of aqueous 0.5% (v/v) acetic acid. The eluate was passed through a 0.2-μm filter and analyzed by LC–MS/MS coupled to an AQUASIL C18 column. For the quality control of laboratory procedures, we processed one procedural blank in each batch of 20 samples. The average recoveries and relative standard deviations (RSDs) of target metabolites in spiked samples ranged from 75.2% to 110.5% and from 0.5% to 9.9% at 10 ng/mL, respectively. Limits of detection (LODs) were established at a signal-to-noise ratio (S/N) of 3:1. The method had LODs of 0.179, 0.344, 0.117, 0.117, 0.417, 0.003, 0.033, 0.033, 0.017 and 0.020 μg/L for MMP, MEP, MnBP, MiBP, MBzP, MEHP, MEOHP, MECPP, MEHHP, and MCMHP, respectively. The LOD was ranged from 0.003 to 0.417 ng/mL.

### 2.4. Measurement of Sex Hormones and Thyroid Hormones 

At the time of delivery, a blood sample (10–30 mL) was collected from the umbilical cord and stored at −80°C until analysis. Free testosterone (FT), estradiol (E2), thyroid stimulating hormone (TSH), total and free thyroxine (T4 and FT4) and triiodothyronine (T3 and FT3) concentrations were measured by an enzyme-linked immunosorbent assay (ELISA) (Shanghai Lianshuo Biological Technology Co, Ltd) according to the manufacturer’s instructions. 

### 2.5. Statistical Analysis

All statistical analysis was performed using SPSS software (version 22.0; IBM SPSS, Chicago, IL, USA). In the descriptive analysis, creatinine-adjusted concentrations were skew distributed and therefore presented as minimums, geometric means (GMs), percentiles, medians and maximums. The concentrations of phthalate metabolites below the LOD were replaced with 1/2 LOD [33]. 

The skew-distributed concentrations of metabolites were log-transformed before performing statistical analyses. A multiple linear regression model was used to analyze the association of urinary concentrations of phthalate metabolites with 7 birth size parameters and gestational age. The logistic regression analyses were applied to explore the relationships between categorical variables and urinary concentrations of phthalate monoesters. In the regression models, the ln-transformed monoester concentrations were used as independent variables and the birth size parameters and pregnancy outcomes were used as a dependent variable. Then, an additional stratified analysis was conducted to explore the gender differences. The model included the following covariates: mother age, gestational age, pre-pregnancy body mass index (BMI), gender of newborn and gestational diabetes mellitus. All phthalate metabolites and outcomes followed normal distribution after log-transformation. Statistical significance was defined as a two-tailed *p*-value of <0.05.

## 3. Results 

### 3.1. Population Characteristics

Table 1 and Table 2 present the characteristics of mothers and newborns, respectively. The median (interquartile range) of gestational week was 38.9 (38.0, 40.0). The mothers had a median BMI of 21.7 kg/m^2^, 23.5% of them had PROM, and 13.9% of them had GDM. The newborns consisted of 118 males and 133 females. Significant differences were found between male and female newborns for BPD (*p* = 0.047) and AGD (*p* < 0.001). Male newborns had longer BPD and AGD than female newborns.

### 3.2. The Concentrations of Phthalate Metabolites in Meconium Samples

Table 3 shows the GM and percentile distribution of phthalate metabolites of mothers with male and female newborns, respectively. MiBP, MnBP and MEHP had relatively higher concentrations than other metabolites. Gender difference was found to be significant only in MiBP, MnBP and MEHP. The detection rates of MMP, MEP, MnBP, MiBP, MBzP, MEHP, MEOHP, MECPP, MEHHP and MCMHP were 97.5%, 74.1%, 99.2%, 99.2%, 7.0%, 100%, 61.3%, 59.3%, 79.4% and 82.7%, respectively. Of note, only 7% of samples were able to be detected for MBzP. Additionally, no significant association was found in the subsequent result. Therefore, we did not present the result of MBzP. 

### 3.3. Birth Size Parameters and Pregnancy Outcomes in Association with Meconium Exposure to Phthalates in the Overall Newborns

The associations between the log-transformed concentrations of metabolites and outcomes of the overall newborns are presented in Tables S1–S3. Significant associations were found between BL and MnBP, MiBP and MEHP with regression coefficients (β) ranging from 0.002 to 0.003, and between FL and MEOHP, MEHHP and MECPP with β ranging from 0.002 to 0.006.

### 3.4. Birth Size Parameters and Pregnancy Outcomes in Association with Meconium Exposure to Phthalates in Female and Male Newborns

Table 4 and Table 5 present the estimates from linear regression models fitting outcomes with ln-transformed metabolites of phthalates in male and female newborns. In females, significant associations were found between BL and MnBP (β = 0.003, *p* = 0.002), MiBP (β = 0.003, *p* = 0.002) and MEHP (β = 0.002, *p* = 0.002), and between FL and MEOHP (β = 0.006, *p* = 0.016), MEHHP (β = 0.006, *p* = 0.023) and MCMHP (β = 0.004, *p* = 0.037). In males, significant associations were found between BL and MnBP and MiBP with the same β of 0.003, and between FL and MEOHP (β = 0.005, *p* = 0.032), and between AC and MECPP (β = 0.004, *p* = 0.021), and between gestational age and MnBP (β = 0.009, *p* = 0.029).

Table 6 presents the estimates from logistic regression models fitting GDM and PROM with metabolites of phthalates in male and female newborns. In male, significant associations were found in GDM with MiBP (β = 2.341, *p* = 0.048), and MEHP (β = 3.506, *p* = 0.018), and in PROM with MnBP (β = 2.595, *p* = 0.036). In female, significant association was only found in PROM with MEP (β = 1.863, *p* = 0.047). 

### 3.5. Sex and Thyroid Hormones in Association with Meconium Exposure to Phthalates in the Overall Newborns

The associations between the ln-transformed concentrations of metabolites and hormone parameters in the overall infants are presented in Tables S4 and S5. Significant associations were only found in TSH with MMP (β = −0.031, *p* = 0.029), and MEHP (β = −0.017, *p* = 0.022). 

### 3.6. Sex and Thyroid Hormones in Association with Meconium Exposure to Phthalates in Female and Male Newborns

Table 7 presents the associations between phthalates exposure and hormones (E2 and TSH). In male, significant associations were found in E2 with MMP (β = 0.044, *p* = 0.044), and MiBP (β = 0.046, *p* = 0.031), and in TSH with MEP (β = 0.063, *p* = 0.029). In female, significant association was only found in TSH with MMP (β = −0.036, *p* = 0.033).

## 4. Discussion

The study explored the effects of meconium exposure to phthalates on a series of health endpoints including birth size parameters, pregnancy outcomes, and fetal sex and thyroid hormones.

### 4.1. Meconium Exposure to Phthalates 

Meconium has three major advantages. Firstly, the sample volume of meconium from each baby is typically large enough to be detected for a trace amount of chemicals. Secondly, the meconium sample may provide information on long-term cumulative exposure [34]. Phthalates are ubiquitous in the environment and human bodies. As the fetal development may be affected by in utero exposure to phthalates, evaluation of accumulative exposure is important [8]. Unlike other biological samples, meconium begins to accumulate in the human fetus from 16 weeks of gestation and it is not excreted until delivery [35]. The characteristic of accumulation of chemicals in meconium increases sensitivity of this matrix because of the short half-life of phthalates in other matrices of the human body (e.g., urine). Therefore, meconium may be considered as a more valid dosimeter of prenatal accumulative exposure to phthalates than cord blood and maternal urine [30,34,35]. For example, Zhang et al. (2009) found that MnBP and MEHP had higher levels of concentrations in meconium (1.7 mg/g–5.5 mg/g) than that in cord blood (0.5 mg/L–2.7 mg/L) or maternal blood (0.6 mg/L–2.2 mg/L) [36]. The third is that meconium assesses phthalate exposure more directly related to fetal compartment. Amniotic fluid also has this advantage. Exposure assessment using a matrix closer to the target organs may benefit the analysis on dose-effect associations. 

However, there was also some argument that phthalates were metabolized and excreted primarily via urine [27], and urine sample might serve as a better matrix of in-utero exposure. Non-urine matrices including meconium, blood and amniotic fluid were also questionable since they contained active enzymes (lipases, esterase) that were able to hydrolyze the phthalate diesters from contamination [35]. The controversy of meconium serving as a matrix of exposure limits its use in phthalate assessment. So far, most epidemiological studies used maternal urine, instead of meconium or amniotic fluid that was directly from fetal compartment to assess in-utero exposure to phthalates [37]. It should be noted that the measurement of phthalates in meconium or other fetal-related matrices has the value of proving maternal–fetal–infant transfer of phthalates. Since fetuses are exposed to externally sourced chemicals from their mothers only and the meconium sample is the excreta of fetuses, the fact that the phthalates can be found in the meconium sample probably means that they can be transferred from mother to fetuses. Both our data and several other studies [36,37,38] found high rates of detection on the metabolites of frequently used phthalates, which highlighted the importance of biomonitoring of phthalates in meconium. 

### 4.2. Phthalates, Birth Size Parameters and Pregnancy Outcomes

Phthalate metabolites have been detected so far mostly in urine, cord blood and breast milk [23,25,39]. There is very little information in literature about their presence in meconium. In this study, MiBP, MnBP and MEHP had relatively higher concentrations than other metabolites. Among 5 American meconium samples, the average concentrations of MEOHP and MEHHP were 3.26 and 3.76 ng/g, respectively [30], higher than median concentrations reported here (0.85 and 0.81 ng/g for MEOHP and MEHHP). Another study of five meconium samples reported that only a few other DEHP metabolites were found in meconium [27]. MEHP has been measured in three Chinese studies that all reported very high median concentrations of approximately 3800 μg/g [40], 2900 μg/g [36], and 163.8 μg/g [38], compared to our study (median 65.2 ng/g). Median MnBP concentrations were 1700 μg/g and 101.70 μg/g, versus our study (28.1 ng/g). However, Arbuckle et al. (2016) [26] reported much lower concentrations of MEHP (median 0.64 ng/g), MnBP (2.09 ng/g), MEOHP and MEHHP (0.12 and 0.37 ng/g for MEOHP and MEHHP) than those reported in our data. Of note, many of previous studies had limited sample size, which might be insufficient to provide robust statistical power and undermined the reliability of these results. Thus, confirmation in larger populations is warranted.

The results raised the special health concern on fetuses for the reason of the high exposure. To prevent the adverse health effects on fetuses, it is essential to have a better understanding of the key sources of exposure. In the present study, the study population had relatively high concentrations of MnBP and MiBP. The sources of MnBP and MiBP were multiple (i.e., diet, personal care products, and inhalation) and complicated [32]. In pregnant women, biomonitoring studies that assess prenatal phthalate exposure have found positive associations between the frequent use of personal care products and urinary levels of phthalate metabolites, including MnBP and MiBP [26]. Thus, exposure to these consumer products in daily life may be an important source of fetal exposure. The findings are valuable for establishing optimum regulatory practices in personal care product production.

In this study, we found several potential gender differences. MnBP, MiBP, MEHP and MEOHP were positively associated with BL and FL, which seems more apparent in female newborns. In addition, MMP and MEOHP were positively associated with E2 in male newborns. Some studies have also found some positive associations between phthalates and birth size outcomes. For example, MBzP was positively associated with BL [41] and FL during pregnancy [42]. However, few studies have been conducted to identify the gender differences. Consistent with our results, one study found that more phthalate metabolites appear to have an effect on female infants than on males [43].

Although the importance of sex hormones in fetal development is well-established, the gender-specific effects of anti-androgenic phthalate exposures on the fetal development are still an understudied area. It seems gender may modify the effects of phthalate exposure on birth size outcomes, however, this phenomenon that female infants are more susceptible to phthalates has not been supported by animal studies as well as previous epidemiological studies. Therefore, our findings highlight the need to identify gender-specific effects in epidemiological studies and the role played by other potential mechanism including disruption of the myelination process, lipid metabolism, calcium signaling, oxidative stress, and activation of the peroxisome proliferator-activated receptor (PPAR) [44].

We did not observe consistent associations between meconium exposure to phthalates and reduced gestational age, preterm delivery and PROM. Instead, we found some associations in GDM, which were only apparent in mothers with male newborns. Several studies have reported an association between phthalate metabolite concentrations and diabetes in non-pregnant populations [45,46], finding associations between certain phthalate metabolites and diabetes. Few studies have evaluated the role of phthalates on GDM, and they found some associations between different gestation period exposures to phthalates and impaired glucose tolerance [47]. As far as we know, we are the first study that is trying to look into the association between in-utero exposure to phthalates and GDM. We found the potential gender difference that MnBP, MiBP and MEHP were associated with GDM only in mothers with male newborns. It has been reported that the effect of phthalates on fetal growth and birth outcomes could be different between genders [48,49,50], but no study has reported a gender-specific effect of maternal exposure to phthalates on GDM. One of the possible mechanisms is an action of phthalates through gender or thyroid hormones [51].

### 4.3. Phthalates, Sex and Thyroid Hormones

We did not observe any significant association between meconium exposure to phthalates with FT that has been reported in many studies [42,43]. We only found some significant negative associations between meconium exposure to phthalates and TSH but no other thyroid hormones in the overall population. Therefore, our data could not draw the conclusion that phthalate exposure was associated with FT and thyroid hormones. However, in the gender-stratified analysis, we did find significant positive associations between E2 and MMP and MiBP in males. A study also found higher estradiol concentrations in relation to increased MBzP, MEHP, MEOHP, and MiBP concentrations in early pregnancy [18]. These findings are similar to animal and in vitro studies reporting the estrogenicity of phthalate, and suggest that phthalates may have impacts on estrogen biosynthesis and/or metabolism [18]. However, previous results still differ with respect to the effect of phthalates on estrogen production. DBP exposure during rat gestation was associated with significantly increased postnatal estradiol levels in offspring [52], but in two studies of adult female rodents, circulating estrogen concentrations were reduced following high-dose DEHP exposure [53,54]. Therefore, the impact of phthalate exposure on estrogen steroidogenesis is still not well understood, especially for the gender-specific effect. 

There are several reasons for the inconclusiveness of the association between maternal phthalate exposure and all concerned outcomes here. Firstly, most of the previous studies measured phthalate exposure in cord blood samples, so comparison with those of the present study is difficult. To our knowledge, no study thus far has evaluated meconium measurements in relation to those outcomes. Secondly, this discrepancy may be explained by the fact that the magnitude of the potential endocrine-disrupting effect of phthalates may depend on the timing of exposure during gestation. Thirdly, our meconium exposures may be too low to elicit the inverse effects we hypothesized based on the adverse effect on birth outcomes reported in rodents. It is possible because in these studies exposures were higher than in our population. 

## 5. Limitations

The present study has several major limitations. Firstly, the nature of cross-sectional design cannot confirm the causality. Secondly, in-utero exposure to phthalates was limited to meconium with no data of other matrices, and the association of exposure level between different matrices was therefore not able to be performed. Thirdly, information on lifestyle (e.g., diets and the use of personal care products), body weight gain, placental weight and clinical therapy from mothers that may affect phthalate exposure were not collected and limited the analysis of risk factors. Fourthly, the sample size of our study is also small and cannot rule out the possibility of chance finding. Fifthly, the hydrolyzed metabolites of phthalates may be overestimated because of the contamination with the ubiquitous DEHP.

## 6. Conclusions

This study found some gender-specific associations of meconium exposure to phthalates with GDM, sex hormones and some parameters of birth size. However, the shortcomings of our study limited the explanation of the data. Longitudinal studies using multiple matrices may help to confirm our findings or find new associations. These findings may be critical to help the policy makers establish the threshold of exposure to phthalates at pregnancy.

## Figures and Tables

**Table 1:** Characteristics of the mothers (*n* = 251).

Characteristics	
Age, (GM (IQR), years)	29.0 (27.0, 31.0)
Pre-pregnancy BMI (GM (IQR), kg/m^2^)	21.7 (19.2, 23.5)
Gestational age (GM (IQR), weeks)	38.9 (38.0, 40.0)
Premature rupture of membrane, n (%)	59 (23.5)
Gestational diabetes mellitus, n (%)	35 (13.9)

^*^P < 0.05; GM, geometric mean; IQR, interquartile range.

**Table 2:** Characteristics of the newborns (*n* = 251).

Characteristics	Male (*n* = 118)	Female (*n* = 133)
Birth weight, (GM (IQR), g)	3360.6 (3120.0, 3632.5)	3255.9 (3027.5, 3512.5)
Birth length, (GM (IQR), cm)	50.1 (50.0, 50.0)	50.0 (50.0, 50.0)
Head circumference, (GM (IQR), cm)	34.0 (34.0, 35.0)	34.0 (34.0, 34.0)
Abdominal circumference, (GM (IQR), mm)	321.0 (314.0, 329.8)	318.3 (309.8, 329.0)
Biparietal diameter, (GM (IQR), mm)	91.5 (89.0, 94.0)	90.4 (88.8, 92.0) *
Femur length, (GM (IQR), mm)	68.0 (66.0, 69.0)	68.3 (66.0, 71.0)
Anogenital distance, (GM (IQR), mm)	3.7 (3.0, 5.0)	1.4 (1.0, 2.0) *

* *p* < 0.05; GM, geometric mean; IQR, interquartile range.

**Table 3:** The concentrations of phthalate metabolites in meconium of newborns (*n* = 251, ng/g).

Phthalate Metabolites	Gender ^a^	Percentile					
		Min	25th	50th	75th	Max	GM
MMP	M	0.30	1.71	2.64	4.24	55.0	2.60
	F	0.30	1.45	2.55	4.86	32.10	2.43
MEP	M	0.57	0.57	1.27	2.02	10.60	1.25
	F	0.57	0.57	1.54	2.31	13.50	1.42
MnBP	M	0.19	20.98	26.60	35.5	96.60	26.64
	F	0.19	18.30	28.50	41.05	271.00	18.40
MiBP	M	0.19	15.50	24.10	37.55	224.0	24.05
	F	0.19	19.90	28.60	56.75	281.0	20.31
MBzP	M	0.85	1.19	1.73	3.48	4.93	1.90
	F	1.39	1.64	1.86	2.48	3.70	3.70
MEHP	M	0.02	34.48	67.30	95.65	228.0	51.82
	F	0.02	23.85	55.60	102.75	311.0	26.61
MEOHP	M	0.05	0.05	0.59	0.88	3.78	0.29
	F	0.05	0.05	0.54	0.95	2.67	0.25
MECPP	M	0.002	0.03	0.15	0.84	11.7	0.15
	F	0.004	0.03	0.16	1.00	16.4	0.18
MEHHP	M	0.01	0.05	0.59	1.39	5.37	0.40
	F	2.71	0.05	0.52	1.39	4.58	0.40
MCMHP	M	0.03	0.72	1.31	2.69	38.60	0.92
	F	0.03	0.03	1.32	3.41	93.10	0.76

GM, geometric mean. ^a^ M, mother with male newborns; F, mother with female newborns.

**Table 4:** Linear regression analysis of relationship between the phthalate metabolites and BL and FL in male and female newborns.

Phthalate Metabolites	Female (BL)		Male (BL)		Female (FL)		Male (FL)	
	β (95%CI)	*p*	β (95%CI)	*p*	β (95%CI)	*p*	β (95%CI)	*p*
MMP	0.001 (−0.001, 0.004)	0.346	0.001 (−0.001, 0.004)	0.318	<0.001 (−0.007, 0.007)	0.997	−0.002 (−0.010, 0.005)	0.548
MEP	0.003 (−0.001, 0.007)	0.106	<0.001 (−0.003, 0.003)	0.924	<0.001 (−0.010, 0.010)	0.982	0.004 (−0.004, 0.013)	0.320
MnBP	0.003 (0.001, 0.004)	***0.002***	0.003 (0.001, 0.006)	***0.019***	0.001 (−0.004, 0.005)	0.674	-0.007 (−0.016, 0.003)	0.162
MiBP	0.003 (0.001, 0.004)	***0.002***	0.003 (0.001, 0.006)	***0.005***	<0.001 (−0.008, 0.008)	0.943	<0.001 (−0.005, 0.004)	0.809
MEHP	0.002 (0.002, 0.003)	***0.002***	0.001 (<0.001, 0.003)	0.108	<0.001 (−0.003, 0.003)	0.963	<0.001 (−0.005, 0.006)	0.932
MEOHP	0.001 (−0.001, 0.003)	0.465	<0.001 (−0.002, 0.001)	0.899	0.006 (0.001, 0.011)	***0.016***	0.005 (<0.001, 0.009)	***0.032***
MECPP	<0.001 (−0.002, 0.001)	0.682	<0.001 (−0.001, 0.001)	0.916	0.003 (<0.001, 0.007)	0.080	0.003 (−0.001, 0.006)	0.098
MEHHP	<0.001 (−0.002, 0.002)	0.830	0.001 (−0.001, 0.002)	0.397	0.006 (0.001, 0.011)	***0.023***	0.001 (−0.003, 0.005)	0.538
MCMHP	0.001 (−0.001, 0.002)	0.466	<0.001 (−0.002, 0.001)	0.556	0.004 (<0.001, 0.007)	***0.037***	−0.001 (−0.004, 0.003)	0.768

BL: birth length; FL: femur length. Models were adjusted for covariates including mother’s age, pre-pregnancy body mass index (BMI), gestational age and gestational diabetes mellitus (GDM) status. Bold italic: *p* < 0.05.

**Table 5:** Linear regression analysis of relationship between the phthalate metabolites and AC and gestational age in male and female newborns.

Phthalate Metabolites	Female (AC) ^a^		Male (AC) ^a^		Female (Gestational Age) ^b^		Male (Gestational Age) ^b^	
	β (95%CI)	*p*	β (95%CI)	*p*	β (95%CI)	*p*	β (95%CI)	*p*
MMP	0.001 (−0.005, 0.007)	0.764	−0.005 (−0.013, 0.003)	0.247	0.002 (−0.002, 0.007)	0.315	<0.001 (−0.008, 0.008)	0.977
MEP	0.004 (−0.006, 0.013)	0.446	−0.001 (−0.010, 0.008)	0.868	0.001 (−0.006, 0.007)	0.794	0.001 (−0.008, 0.010)	0.827
MnBP	0.001 (−0.003, 0.005)	0.584	−0.002 (−0.012, 0.008)	0.749	0.002 (−0.001, 0.004)	0.292	0.009 (0.001, 0.017)	***0.029***
MiBP	<0.001 (−0.004, 0.004)	0.928	−0.002 (0.010, 0.006)	0.642	0.001 (−0.002, 0.004)	0.561	0.005 (−0.002, 0.012)	0.186
MEHP	0.001 (−0.002, 0.004)	0.445	0.003 (−0.003, 0.009)	0.251	0.002 (<0.001, 0.003)	0.112	0.005 (<0.001, 0.010)	0.061
MEOHP	0.003 (−0.002, 0.008)	0.205	0.002 (−0.002, 0.007)	0.308	0.002 (−0.022, 0.027)	0.843	0.001 (−0.003, 0.004)	0.713
MECPP	<0.001 (−0.004, 0.003)	0.796	0.004 (0.001, 0.007)	***0.021***	−0.004 (−0.022, 0.013)	0.636	0.001 (−0.001, 0.003)	0.361
MEHHP	0.001 (−0.004, 0.006)	0.638	0.002 (−0.003, 0.006)	0.471	−0.001 (−0.026, 0.023)	0.921	0.001 (−0.003, 0.004)	0.759
MCMHP	0.003 (<0.001, 0.006)	0.056	<0.001 (−0.004, 0.003)	0.797	−0.003 (−0.019, 0.014)	0.748	0.001 (−0.001, 0.004)	0.211

AC: abdominal circumference. ^a^ Models were adjusted for covariates including mother’s age, pre-pregnancy BMI, gestational age and GDM status. ^b^ Models were adjusted for covariates including mother’s age, pre-pregnancy BMI and GDM status. Bold italic: *p* < 0.05.

**Table 6:** Logistic regression analysis of relationship between the phthalate metabolites and GDM and PROM in male and female newborns.

Phthalate Metabolites	Female (GDM)		Male (GDM)		Female (PROM)		Male (PROM)	
	β (95%CI)	*p*	β (95%CI)	*p*	β (95%CI)	*p*	β (95%CI)	*p*
MMP	1.594 (0.920, 2.761)	0.096	1.533 (0.776, 3.107)	0.213	1.040 (0.699, 1.548)	0.845	1.430 (0.829, 2.468)	0.198
MEP	1.703 (0.826, 3.514)	0.149	1.396 (0.394, 4.949)	0.605	1.863 (1.009, 3.438)	***0.047***	1.146 (0.589, 2.230)	0.688
MnBP	1.454 (0.835, 2.533)	0.186	3.102 (0.869, 11.207)	0.084	0.984 (0.747, 1.295)	0.906	2.595 (1.062, 6.344)	***0.036***
MiBP	1.237 (0.819, 1.869)	0.311	2.341 (1.009, 5.432)	***0.048***	1.194 (0.879, 1.622)	0.257	1.121 (0.652, 1.927)	0.680
MEHP	1.304 (0.877, 1.937)	0.189	3.506 (1.239, 9.924)	***0.018***	1.176 (0.922, 1.500)	0.192	1.326 (0.848, 2.074)	0.216
MEOHP	0.979 (0.674, 1.422)	0.910	2.794 (0.516, 15.135)	0.233	1.146 (0.839, 1.564)	0.391	1.142 (0.815, 1.599)	0.441
MECPP	1.029 (0.793, 1.336)	0.829	1.148 (0.685, 1.925)	0.600	1.031 (0.831, 1.278)	0.784	1.108 (0.860, 1.426)	0.428
MEHHP	0.863 (0.600, 1.240)	0.425	1.145 (0.602, 2.176)	0.680	1.059 (0.777, 1.445)	0.715	1.023 (0.754, 1.387)	0.885
MCMHP	1.018 (0.794, 1.305)	0.890	1.364 (0.755, 2.462)	0.304	1.078(0.876, 1.327)	0.477	1.170 (0.884, 1.549)	0.273

GDM: gestational diabetes; PROM: premature rupture of membrane. Models were adjusted for covariates including mother’s age, pre-pregnancy BMI and gestational age. Bold italic: *p* < 0.05.

**Table 7:** Linear regression analysis of relationship between the phthalate metabolites and sex and thyroid hormones in cord blood.

Phthalate Metabolites	Female (E2)		Male (E2)		Female (TSH)		Male (TSH)	
	β (95%CI)	*p*	β (95%CI)	*p*	β (95%CI)	*p*	β (95%CI)	*p*
MMP	0.007 (−0.025, 0.039)	0.668	0.044 (0.001, 0.086)	***0.044***	−0.036 (−0.069, 0.003)	***0.033***	−0.017 (−0.069, 0.035)	0.520
MEP	0.008 (−0.040, 0.055)	0.749	0.023 (−0.025, 0.071)	0.340	−0.011 (−0.060, 0.039)	0.673	0.063 (0.007, 0.120)	***0.029***
MnBP	0.007 (−0.015, 0.029)	0.538	0.026 (−0.027, 0.080)	0.333	−0.018 (−0.041, 0.004)	0.112	−0.011 (−0.076, 0.053)	0.728
MiBP	0.010 (−0.011, 0.031)	0.329	0.046 (0.004, 0.088)	***0.031***	−0.018 (−0.039, 0.004)	0.111	−0.005 (−0.056, 0.047)	0.860
MEHP	0.007 (−0.008, 0.022)	0.363	−0.005 (−0.037, 0.027)	0.759	−0.013 (−0.028, 0.002)	0.097	−0.034 (−0.072, 0.004)	0.075
MEOHP	0.002 (−0.022, 0.027)	0.843	0.021 (−0.003, 0.046)	0.081	0.020 (−0.006, 0.046)	0.123	0.007 (−0.023, 0.036)	0.654
MECPP	−0.004 (−0.022, 0.013)	0.636	0.008 (−0.011, 0.026)	0.413	0.009 (−0.009, 0.028)	0.321	−0.006 (−0.029, 0.016)	0.577
MEHHP	−0.001 (−0.026, 0.023)	0.921	0.016 (−0.006, 0.038)	0.159	−0.017 (−0.043, 0.008)	0.179	0.015 (−0.011, 0.042)	0.250
MCMHP	−0.003 (−0.019, 0.014)	0.748	0.012 (−0.008, 0.031)	0.238	−0.004 (−0.022, 0.013)	0.625	−0.012 (−0.036, 0.011)	0.291

E2: estradiol; TSH: thyroid stimulating hormone. Models were adjusted for covariates including mother’s age, gestational age, pre-pregnancy BMI and GDM status. Bold italic: *p* < 0.05.

## Supplementary Materials

The following are available online at https://www.mdpi.com/1660-4601/17/21/7711/s1, Table S1: Linear regression analysis of relationship between the phthalate metabolites and body size parameters in overall newborns, Table S2: Linear regression analysis of relationship between the phthalate metabolites and body size parameters in overall newborns, Table S3: Regression analysis of relationship between the phthalate metabolites and pregnancy outcomes in overall newborns, Table S4: Linear regression analysis of relationship between the phthalate metabolites and hormones parameters in in overall newborns, Table S5: Linear regression analysis of relationship between the phthalate metabolites and hormones parameters in in overall newborns.
