# Supplementary material for: Meconium Exposure to Phthalates, Sex and Thyroid Hormones, Birth Size and Pregnancy Outcomes in 251 Mother–Infant Pairs from Shanghai
Source: Int J Environ Res Public Health. 2020 Oct 22;17(21):7711. doi: 10.3390/ijerph17217711 (PMC7659924; doi:10.3390/ijerph17217711)
Supplement: Supplementary file 1 [file ijerph-17-07711-s001.pdf]

Table S1. Linear regression analysis of relationship between the phthalate metabolites and body size parameters in overall newborns.

Phthalate metabolites	BW (g)		BL (cm)		AC(mm)		AGD(mm)	
	β (95%CI)	*P*	β (95%CI)	*P*	β (95%CI)	*P*	β (95%CI)	*P*
MMP	-0.004 (-0.016,0.008)	0.464	0.002 (-0.001,0.004)	0.175	-0.001 (-0.006,0.004)	0.778	0.021 (-0.049,0.091)	0.559
MEP	0.004 (-0.012,0.020)	0.607	0.003 (<0.001,0.006)	0.075	0.002 (-0.005,0.008)	0.609	0.014 (-0.077,0.105)	0.761
MnBP	-0.008 (-0.017,0.001)	0.091	0.003 (0.001,0.004)	**<0.001**	0.001 (-0.003,0.005)	0.673	-0.011 (-0.063,0.040)	0.665
MiBP	-0.007 (-0.016,0.001)	0.100	0.003 (0.001,0.004)	**<0.001**	<0.001 (-0.004,0.003)	0.926	0.013 (-0.036,0.061)	0.609
MEHP	-0.005 (-0.010,0.001)	0.131	0.002 (0.001,0.003)	**<0.001**	0.001 (-0.001,0.006)	0.118	-0.009 (-0.044,0.026)	0.610
MEOHP	0.005 (-0.003,0.014)	0.205	0.001 (-0.001,0.002)	0.244	0.003 (-0.003,0.002)	0.539	0.045 (-0.001,0.091)	0.055
MECPP	0.002 (-0.004,0.008)	0.473	<0.001 (-0.001,0.001)	0.842	<0.001 (-0.005,0.004)	0.834	0.026 (-0.008,0.060)	0.129
MEHHP	0.003 (-0.005,0.011)	0.410	0.001 (-0.001,0.002)	0.229	0.001 (-0.002,0.004)	0.437	0.028 (-0.015,0.071)	0.197
MCMHP	0.001 (-0.005,0.007)	0.851	<0.001 (-0.001,0.001)	0.493	0.002 (-0.001, 0.004)	0.201	0.024 (-0.009,0.057)	0.151

BW: birth weight; BL: birth length; AC: abdominal circumference; AGD: anogenital distance;

Models were adjusted for covariates including mother’s age, pre-pregnancy BMI, gestational age, sex of newborn, and GDM status.

Bold italic: $P < 0.05$

Table S2. Linear regression analysis of relationship between the phthalate metabolites and body size parameters in overall newborns.

Phthalate metabolites	HC (mm)		BPD (mm)		FL (mm)	
	β (95%CI)	*P*	β (95%CI)	*P*	β (95%CI)	*P*
MMP	-0.002 (-0.006,0.002)	0.429	0.001 (-0.004,0.005)	0.771	<0.001 (-0.005,0.005)	0.892
MEP	-0.002 (-0.008,0.003)	0.391	-0.001 (-0.007,0.005)	0.707	0.002 (-0.004,0.009)	0.498
MnBP	0.001 (-0.002,0.004)	0.494	0.002 (-0.002,0.005)	0.308	<0.001 (-0.003,0.004)	0.803
MiBP	<0.001 (-0.003,0.003)	0.878	0.002 (-0.002,0.005)	0.351	<0.001 (-0.004,0.003)	0.913
MEHP	0.001 (-0.001,0.003)	0.351	0.001 (-0.001,0.004)	0.257	0.001 (-0.002,0.003)	0.673
MEOHP	<0.001 (-0.003,0.003)	0.993	<0.001 (-0.003, 0.003)	0.822	0.006 (0.002,0.009)	**0.001**
MECPP	0.001 (-0.001,0.003)	0.170	0.002 (-0.001,0.004)	0.173	0.003 (0.001,0.006)	**0.013**
MEHHP	<0.001 (0.002,0.015)	0.868	-0.001 (-0.004,0.002)	0.451	0.003 (<0.001,0.007)	**0.034**
MCMHP	0.002 (<0.001,0.004)	0.057	0.001 (-0.002,0.003)	0.542	0.002 (<0.001,0.005)	0.053

HC: head circumference; BPD: biparietal diameter; FL: femur length.

Models were adjusted for covariates including mother’s age, pre-pregnancy BMI, gestational age, sex of newborn, and GDM status.

Bold italic: *P* < 0.05

Table S3. Regression analysis of relationship between the phthalate metabolites and pregnancy outcomes in overall newborns.

Phthalate metabolites	GDM (mm)		PROM (mm)		Gestational age (mm)	
	β (95%CI)	*P*	β (95%CI)	*P*	β (95%CI)	*P*
MMP	1.382 (0.926,2.062)	0.113	1.165 (0.845,1.606)	0.350	0.001 (-0.003,0.005)	0.533
MEP	1.139 (0.687,1.889)	0.614	1.466 (0.948,2.266)	0.085	<0.001 (-0.005,0.006)	0.941
MnBP	1.230 (0.838,1.804)	0.290	1.112 (0.843,1.467)	0.451	0.002 (<0.001,0.005)	0.101
MiBP	1.214 (0.864,1.707)	0.263	1.193 (0.912,1.561)	0.198	0.001 (-0.001,0.004)	0.322
MEHP	1.149 (0.892,1.482)	0.282	1.206 (0.970,1.500)	0.093	0.002 (<0.001,0.004)	**0.045**
MEOHP	0.907 (0.698,1.179)	0.467	1.122 (0.895,1.406)	0.318	0.001 (-0.002,0.003)	0.675
MECPP	1.013 (0.836,1.226)	0.897	1.053 (0.895,1.239)	0.533	0.001 (-0.001,0.003)	0.602
MEHHP	0.890 (0.002,0.015)	0.350	1.050 (0.848,1.301)	0.652	0.001 (-0.002,0.003)	0.538
MCMHP	1.066 (0.879,1.294)	0.515	1.110 (0.943,1.307)	0.211	0.002 (<0.001,0.004)	0.126

GDM: gestational diabetes; PROM: premature rupture of membrane;

Models were adjusted for covariates including mother's age, pre-pregnancy BMI, gestational age, sex of newborn, and GDM status.

Bold italic: $P < 0.05$

Table S4. Linear regression analysis of relationship between the phthalate metabolites and hormones parameters in in overall newborns.

Phthalate metabolites	E2		FT		TSH	
	β (95%CI)	*P*	β (95%CI)	*P*	β (95%CI)	*P*
MMP	0.017 (-0.008,0.042)	0.184	0.007 (-0.019,0.033)	0.589	-0.031 (-0.059,-0.003)	**0.029**
MEP	0.015 (-0.019,0.048)	0.388	-0.003 (-0.036,0.031)	0.876	0.023 (-0.014,0.060)	0.226
MnBP	0.010 (-0.010,0.029)	0.321	0.009 (-0.010,0.029)	0.345	-0.018 (-0.039,-0.004)	0.104
MiBP	0.017 (-0.001,0.035)	0.058	0.005 (-0.013,0.024)	0.562	-0.015 (-0.036,0.005)	0.131
MEHP	0.004 (-0.008,0.017)	0.505	0.003 (-0.010,0.016)	0.674	-0.017 (-0.031,-0.002)	**0.022**
MEOHP	0.011 (-0.006,0.028)	0.198	-0.005 (-0.022,0.013)	0.590	0.014 (-0.005,0.033)	0.160
MECPP	<0.001 (-0.012,0.013)	0.954	-0.009 (-0.021,0.004)	0.193	0.002 (-0.012,0.016)	0.786
MEHHP	0.008 (-0.008,0.024)	0.339	0.015 (-0.001,0.032)	0.072	<0.001 (-0.019,0.018)	0.970
MCMHP	0.001 (-0.001,0.014)	0.838	-0.003 (-0.015,0.010)	0.649	-0.008(-0.022,0.006)	0.252

E2: estradiol; FT: free testosterone; TSH: thyroid stimulating hormone;

Models were adjusted for covariates including mother’s age, pre-pregnancy BMI, gestational age, sex of newborn, and GDM status.

Bold italic: $P < 0.05$

Table S5. Linear regression analysis of relationship between the phthalate metabolites and hormones parameters in in overall newborns.

Phthalate metabolites	T3		T4		FT3		FT4	
	β (95%CI)	*P*	β (95%CI)	*P*	β (95%CI)	*P*	β (95%CI)	*P*
MMP	0.003 (-0.025,0.031)	0.845	0.003 (-0.028,0.034)	0.849	0.017 (-0.010,-0.044)	0.214	-0.025 (-0.056,0.005)	0.100
MEP	0.002 (-0.035,0.039)	0.922	0.007 (-0.033,0.048)	0.723	0.014 (-0.022,0.050)	0.431	-0.035 (-0.075,0.005)	0.083
MnBP	<0.001 (-0.021,0.022)	0.979	-0.004 (-0.027,0.020)	0.761	0.005 (-0.016,-0.026)	0.671	-0.014 (-0.037,0.009)	0.241
MiBP	0.003 (-0.017,0.023)	0.779	-0.003 (-0.025,0.019)	0.765	0.007 (-0.012,0.027)	0.456	-0.011 (-0.033,0.011)	0.307
MEHP	-0.003 (-0.017,0.011)	0.675	<0.001 (-0.016,0.015)	0.959	0.006 (-0.008,-0.020)	0.377	-0.010 (-0.026,0.005)	0.192
MEOHP	0.010 (-0.009,0.029)	0.293	-0.001 (-0.022,0.021)	0.956	0.001 (-0.020,0.017)	0.883	-0.003 (-0.024,0.018)	0.766
MECPP	-0.003 (-0.017,0.011)	0.713	0.005 (-0.010,0.021)	0.522	-0.004 (-0.018,0.010)	0.558	-0.002 (-0.018,0.013)	0.765
MEHHP	<0.001 (-0.018,0.018)	0.981	0.009 (-0.011,0.029)	0.375	-0.008 (-0.026,0.009)	0.362	-0.008 (-0.027,0.012)	0.442
MCMHP	0.003 (-0.011,0.017)	0.685	0.002 (-0.014,0.017)	0.841	-0.006 (-0.019,0.008)	0.396	-0.002 (-0.017,0.013)	0.821

T3: total triiodothyronine; FT3: free triiodothyronine; T4: total thyroxine; FT4: free thyroxine;

Models were adjusted for covariates including mother’s age, pre-pregnancy BMI, gestational age, sex of newborn, and GDM status.

Bold italic: $P < 0.05$
